# Searching for signatures of positive selection in cytochrome* b* gene associated with subterranean lifestyle in fast-evolving arvicolines (Arvicolinae, Cricetidae, Rodentia)

**DOI:** 10.1186/s12862-021-01819-4

**Published:** 2021-05-20

**Authors:** Olga V. Bondareva, Nadezhda A. Potapova, Kirill A. Konovalov, Tatyana V. Petrova, Natalia I. Abramson

**Affiliations:** 1 Zoological Institute RAS, Saint-Petersburg, Russia; 2 Institute for Information Transmission Problems (Kharkevich Institute) RAS, Moscow, Russia; 3 Hong Kong University of Science and Technology, Hong Kong, Hong Kong

**Keywords:** Adaptation, Subterranean lifestyle, Cytochrome *b*, Arvicolinae, Natural selection

## Abstract

**Background:**

Mitochondrial genes encode proteins involved in oxidative phosphorylation. Variations in lifestyle and ecological niche can be directly reflected in metabolic performance. Subterranean rodents represent a good model for testing hypotheses on adaptive evolution driven by important ecological shifts. Voles and lemmings of the subfamily Arvicolinae (Rodentia: Cricetidae) provide a good example for studies of adaptive radiation. This is the youngest group within the order Rodentia showing the fastest rates of diversification, including the transition to the subterranean lifestyle in several phylogenetically independent lineages.

**Results:**

We evaluated the signatures of selection in the mitochondrial cytochrome *b* (*cytB*) gene in 62 Arvicolinae species characterized by either subterranean or surface-dwelling lifestyle by assessing amino acid sequence variation, exploring the functional consequences of the observed variation in the tertiary protein structure, and estimating selection pressure. Our analysis revealed that: (1) three of the convergent amino acid substitutions were found among phylogenetically distant subterranean species and (2) these substitutions may have an influence on the protein complex structure, (3) *cytB* showed an increased ω and evidence of relaxed selection in subterranean lineages, relative to non-subterranean, and (4) eight protein domains possess increased nonsynonymous substitutions ratio in subterranean species.

**Conclusions:**

Our study provides insights into the adaptive evolution of the cytochrome *b* gene in the Arvicolinae subfamily and its potential implications in the molecular mechanism of adaptation. We present a framework for future characterizations of the impact of specific mutations on the function, physiology, and interactions of the mtDNA-encoded proteins involved in oxidative phosphorylation.

**Supplementary Information:**

The online version contains supplementary material available at 10.1186/s12862-021-01819-4.

## Background

Understanding mechanisms involved in the formation of adaptations at the molecular level is among the main challenges of evolutionary biology. The occurrence of similar adaptations in pairs of phylogenetically related or distant organisms provides a good opportunity to test hypotheses about the mechanisms of natural selection in evolution at the molecular level.

Subterranean rodents represent a good model for testing hypotheses on adaptive evolution driven by important ecological shifts. They live in a subterranean environment characterized by high levels of carbon dioxide, low levels of oxygen, and a relatively constant temperature and humidity [1]. Being subjected to the drastic change in energy requirements [2] associated with colonization of the subterranean niche, in particular the transition from oxygen-rich to hypoxic atmospheres [3, 4], it was supposed that selective regimes of the proteins involved in respiration may have experienced positive directional selection in response to their entry into the subterranean habitat [5].

Voles and lemmings of the subfamily Arvicolinae are remarkable as it is the youngest group among rodents, is rapidly evolving, and is one of the most diverse groups that have colonized almost all landscapes and habitat types in the Northern Hemisphere. Arvicolinae display the fastest documented adaptive radiation among modern mammals. The earliest arvicolids are known from the Late Miocene (ex. gr. 7–8 Ma) both in Eurasia and North America [6, 7]. The subfamily consists of about 150 species grouped according to various opinions into 28–30 genera belonging to 8–10 tribes [8]. The number of extant arvicoline rodents is eight times greater than in the sister taxon Cricetinae, the most recent common ancestors (MRCA) of both are known in the fossil record from the Late Miocene, ca 10 Ma [7]. Arvicolinae emerged during the series of repeated events of rapid speciation with at least three “explosive” periods of rapid divergence during its evolutionary history [9].

Within this group, at least 5 phylogenetically distant lineages counting altogether nearly 10 species show independent transition to the subterranean lifestyle. The long-clawed mole vole, *Prometheomys schaposchnikowi* Satunin, 1901 represents the earliest evolutionary lineage among all recent arvicolids and is the only subterranean form among the so-called first radiation of voles [9]. This early split between *Prometheomys* and all other voles according to molecular dating was estimated around 7 Ma [9, 10]. Other subterranean lineages appear much later and are related to the most speciose and recent radiation wave. Among these lineages are the highly specialized subterranean mole voles of the tribe Ellobiusini with the only genus *Ellobius* Fisher, 1814 that counts 5 species in two subgenera. Fossil remains of mole voles are known from the boundary of Pliocene–Pleistocene, approximately 2.5 Ma [11, 12], molecular dating estimates of the mole voles lineage split are approx. 4.5–4.8 Ma [9, 13]. Several species from different genera within the most diverse and species-rich tribe Arvicolini (encountering 60–62 species) also show the various extent of adaptation to subterranean lifestyle, particularly: *Terricola subterraneus* de Selys-Longshamps, 1836, *Microtus pinetorum* Le Conte 1830 and *Lasiopodomys mandarinus* Milne-Edwards, 1830. These species belong to different nodes within the tribe [14] and are not descendants of the MRCA. The sister taxa of each species are surface-dwelling which indicate the independent multiple transitions to subterranean lifestyle within this tribe. The basal radiation of all microtines (including and *Lasiopodomys*) crown lineages may be estimated as 2.1 Ma so far as according to known fossil record multiple rootless forms assigned to *Microtus* appear throughout the Northern Hemisphere at 1.9–2.1 Ma [15, 16]. Despite this ecological and phylogenetical diversity, molecular signatures of selection associated with mastering various ecological niches, including subterranean environment are poorly studied.

Mitochondrial genes have often been assumed to be under strong purifying selection because they encode proteins involved in oxidative phosphorylation (OXPHOS) that can directly influence metabolic performance. However, lifestyle shifts that imply an alteration in metabolism might be associated with changes in the selection pressure of those proteins that participate in the biochemical pathways of cellular respiration. Cytochrome *b* (*cytB*) is a key component of *bc*_*1*_, one of the protein complexes involved in oxidative phosphorylation in the mitochondrial membrane. It catalyzes the reversible electron transfer from ubiquinol to cytochrome *c* coupled to proton translocation (Q-cycle [17, 18]). Despite *cytB* having been extensively used for phylogenetic and phylogeographic studies as a neutral evolutionary marker, many studies provide support for instances of adaptive selection in mammalian mitochondrial protein-coding genes and *cytB*. After [5] several papers gave evidence in favor of signatures of positive selection in the evolution of this gene [19–21]. Subsequent studies have suggested that, despite strong functional constraints, mtDNA may be subjected to positive directional selection in cases, for example, of energy-demanding lifestyles and/or the limited availability of oxygen [22–24]. In turn, Nevo [25] correlated sequence variation of a portion of the *cytB* gene with ecological differences between chromosomal races of blind mole-rats *Spalax ehrenbergi Nehring, 1897*. To date, the influence of these variations on respiratory function remains unclear. Da Silva et al. [5] found a significantly higher estimated dN/dS ratio (ω)—the ratio of nonsynonymous to synonymous substitutions—in independent lineages of subterranean rodents concerning their non-subterranean counterparts, suggesting a link between the evolution of this gene and the colonization of a hypoxic environment. This observation was later confirmed [24] using a set of seven complete mtDNA genomes of octodontoids.

As *cytB* is an important component of the electron transport chain and thus respiratory processes of the cell, several attempts have also been implemented to find adaptive amino acid changes in partial and complete sequences of this gene. Some amino acid changes may improve aerobic capacity and adaptations to new thermal environments [22, 26–29]. For instance, mutations in mitochondrial genes have been implicated in exercise intolerance in humans [28]. Da Silva et al. [5] detected many amino acid substitutions in the *cytB* sequence in the tuco-tucos, coruro, pocket gophers and African mole rats, in both unique and in the same positions for all species.

In this study, we analyzed the between and within species variation of the *cytB* gene among subterranean vole species, with special reference to signatures of positive selection on the background of a wider group of surface-dwelling rodents from the Arvicolinae subfamily.

Earlier, an acceleration of substitution rates in *cytB* in subterranean rodents was shown [5]; however, authors carried out their analysis in a phylogenetic framework at the family level and over a large evolutionary period. Here, we tested the hypothesis of whether the phenomenon [5] described is true at a significantly smaller evolutionary scale and within a lower taxonomic level. Comparing different genera within subfamily and different species within one genus that independently mastered the subterranean lifestyle, we aimed to determine whether the transition to the subterranean life is followed by positive selection in *cytB* and whether the substitutions occur at homologous or different sites.

## Results

**Fig. 1:**
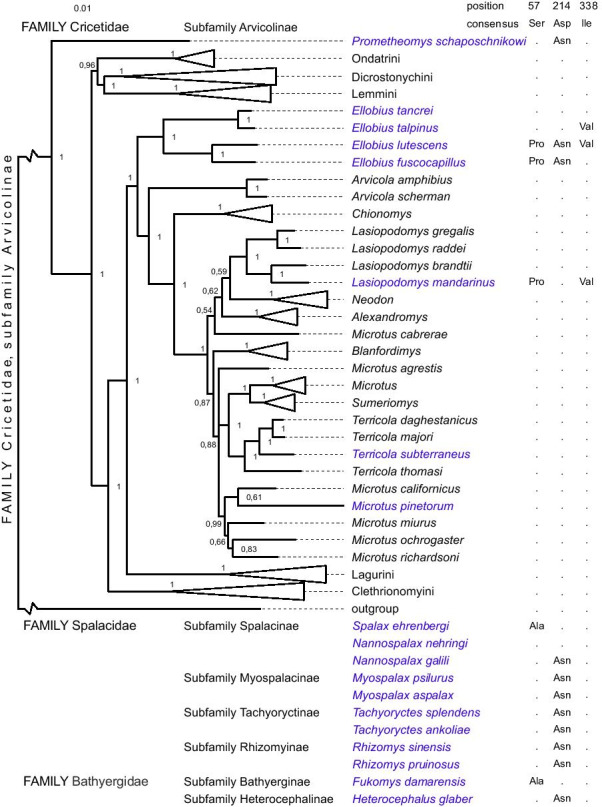
Phylogenetic tree with observed substitutions unique for subterranean species. Fifty percent majority rule consensus tree from Bayesian inference analysis. Numbers are Bayesian posterior probabilities. Subterranean species are shown in blue. The size of the triangles corresponding to tribes and genera is proportional to the number of species sequenced. On the right, the amino acid substitutions in certain positions are shown. Below the tree, the amino acid substitutions in the same sites in specialized subterranean rodents from other families are shown

In this study, we search the natural selection traces in subterranean species of the Arvicolinae subfamily. We analyzed sequences of protein-coding mitochondrial gene cytochrome *b* for 62 species shown on Fig. 1. Our study covers representatives of all main taxonomic groups and genera that allow us to study species with various times of divergence. We obtained a list of TreeSAAP significant substitutions (categories 6–8) for the studied species in a phylogenetic context since the sequences were analyzed with an account of the phylogenetic position of the species on the tree. From the list of significant substitutions, we selected those typical of at least 3 subterranean species. We found three substitutions that satisfied our criteria: Ser57Pro, Asp214Asn and Ile338Val (Fig. 1). The same substitution type Asp214Asn was also found in specialized subterranean rodents belonging to other families.

Comparison of amino acid frequencies per site revealed (amino acid patterns) more than 80 sites being significantly different between subsets of subterranean and non-subterranean species (Additional files 1, 2b). The substitutions 57 and 338 were among these.

The serine to proline substitution at residue 57 in subterranean rodents potentially removes a phosphorylation site. We used two different methods to predict the phosphorylation of this site. NetPhos 3.1 Server predicted phosphorylation with CDC2 kinase with score 0.518, GPS 5.0 predicted AGC, PKN, PKN1 kinases with score 65.363. The predictions of the type of kinase do not agree with each other, however, all predictions assign high probabilities to this site being phosphorylated. The same methods did not predict phosphorylation for Asp214, and to our knowledge, neither Ile nor Val can be phosphorylated.

A single nucleotide polymorphism at position 338 (ATT > GTT) corresponding to Ile338Val substitution was detected as likely pathogenic in the ClinVar NCBI database and is associated with cancer processes: www.ncbi.nlm.nih.gov/clinvar/variation/143898/.

**Fig. 2:**
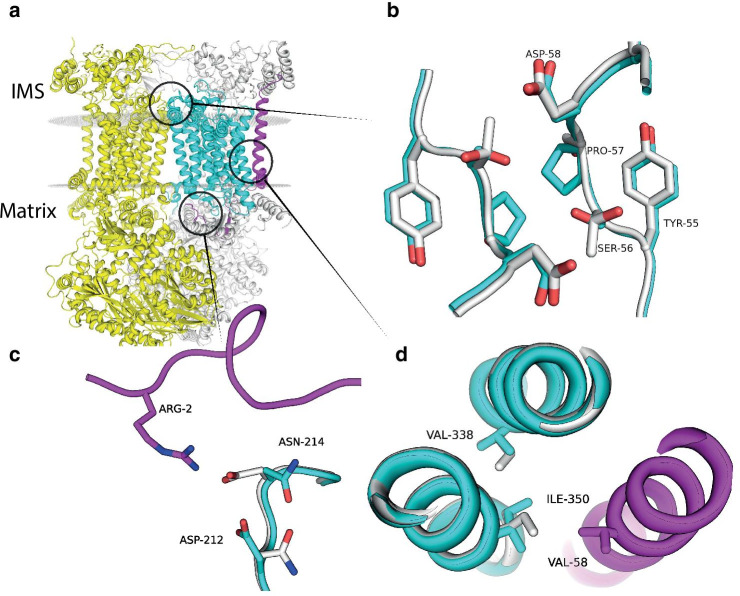
Structural model of substitutions in the cytochrome *bc*_*1*_ complex. **a** Overview of the cytochrome *bc*_*1*_ homodimer. Cytochrome *b* is cyan, UQCRQ is magenta. The second monomer of the complex is colored yellow. The substitution locations are highlighted with circles. *IMS* intermembrane space. **b** Overlaid structures of *E. lutescens* and *L. sibiricus* showing the Ser57Pro substitution. *E. lutescens* model is cyan, *L. sibiricus* is white. **c** The Asp214Asn substitution and its interaction with the N-terminus UQCRQ (magenta) **d** The Ile338Val substitution and the neighboring UQCRQ chain (magenta)

We modeled the structure of *cytB* to examine the possible effect of three substitutions: Ser57Pro, Asp214Asn, and Ile338Val (Fig. 2a). Based on our model, Ser57 faces the intermembrane space of the mitochondria. It resides on an unstructured loop segment spanning residues 54–60. This loop contacts the same loop on the second cytochrome *bc*_*1*_ monomer in the complex (Fig. 2b). Unlike Ser57Pro, substitution Asp214Asn is located on a loop facing the matrix of the mitochondrion. It contacts the N-terminus of ubiquinol-cytochrome *c* reductase complex III subunit VII (UQCRQ) (Fig. 2c). Substitution Ile338Val is located on the interface between α-helices in the transmembrane region of the complex (Fig. 2d). The modeled structure shows that this substitution favors a different rotamer of Ile350, which neighbors residue 58 of UQCRQ.

We observe a tendency towards weakening purifying selection in subterranean rodents in branch analysis. All species showed significant differences compared with the neutral model (b_neut [31]) on branches by LRT (Table 1) that indicate the signature of relaxe selection in *cytB* gene. Significant differences between foreground branches (subterranean species) and background branches (surface-dwelling species) were indicated for *Ellobius* species, *Lasiopodomys mandarinus* and *Terricola subterraneus* according to LRT results comparing free-ratio (b_free) and one ratio (M0) models. The same result was obtained in the analysis total set of subterranean species.

**Table 1:** Estimation of ω in ete-toolkit using a branch model and RELAX analysis results

	branch model analysis				RELAX		
Subterranean species	Fg, ω1	Bg, ω0	P_LRT b_free and M0	P_LRT b_free and b_neut	K	P	LRT
*All species*	**0.0566**	**0.0303**	**2.45E-06**	**1.46E-125**	N/A	N/A	N/A
*Ellobius sp.*	**0.0642**	**0.0243**	**2.33E-06**	**4.76E-81**	**0.52**	**0**	**23.97**
*L. mandarinus*	**0.1325**	**0.0269**	**4.69E-05**	**0.00024**	**0**	**0.001**	**11.78**
*M. pinetorum*	0.0233	0.0224	0.9327	**4.62E-24**	1.05	0.804	0.06
*P. schaposchnikowi*	0.0617	0.0356	0.0711	**1.26E-09**	**0.63**	**0.023**	**5.15**
*T. subterraneus*	**0.0071**	**0.0343**	**0.05**	**2.48E-17**	**23.22**	**0.014**	**6.02**

**Fig. 3:**
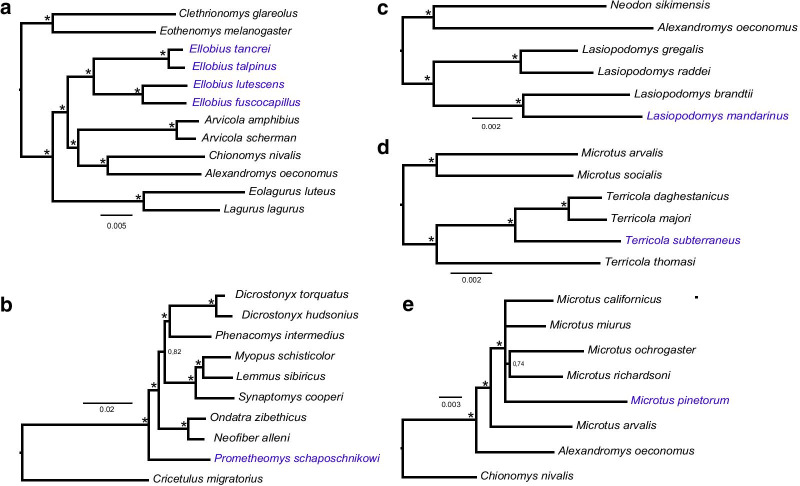
Trees for selection estimation in different branches. Fifty percent majority rule consensus tree from Bayesian inference analysis. Subterranean species are shown in blue. Stars (*) indicated 0.95–1.0 Bayesian posterior probabilities

*Fg* foreground branch (subterranean species), *Bg* background branch (surface-dwelling species). Subterranean species indicated by color on Fig. 3. *P_LRT* likelihood ratio test p-value for models comparison, *M0* one-ratio model, *b_fee* free-branch model, *b_neut* neutral-branch model, *K* selection intensity parameter, *P* p-value, *LRT* likelihood ratio test, *N/A* not analyzed. Significant values indicated by **bold**

Branch-site analysis implemented in the Datamonkey server (aBSREL) found no evidence of episodic diversifying selection in analyzed phylogeny. RELAX confirmed changes in natural selection level of subterranean rodents. So, K-values for three species (*Ellobius sp., L. mandarinus* and *P. schaposchnikowi*; Table 1) demonstrated values < 1, which could indicate relaxed selection. Two of these species: *Ellobius sp.* and *L. mandarinus,* showed increased ω-values in branch analysis. K for *T. subterraneus* demonstrated a value much greater than 1 that could be interpreted as “selection strength has been intensified and correlated with lower ω-value in comparison with surface-dwelling species.

Furthermore, the calculation of the distribution of nucleotide substitutions at each site of *cytB* separately and combined by domain coordinates manually showed the following: site analysis indicated four positions (Table 2) with significantly higher values of substitutions in *cytB* in subterranean species (sites 4, 236, 237, and 241). The search of sites under positive selection, applying sites and branch-sites models, with programs: PAML (site model M2), FEL and MEME, taking into account all species and several clades separately has shown both unique and similar sites in different species (Table 2). The detected positions were further checked for variation in amino acid substitution pattern at intraspecific level (Additional file 1).

**Table 2:** Sites in *cytB* gene under positive selection

	PAML	MEME	FEL	Manual	AA patterns
All species	No sites	234, 249	241	4, 236, 237, 241	4, 234, 237, 241, 249
*Ellobius* sp.	No sites	No sites	241	N/A	241
*L. mandarinus*	42, 238, 241	238	244, 329	N/A	42, 238, 241, 329
*M. pinetorum*	No sites	313	60, 299	N/A	60, 299
*P. schaposchnikowi*	No sites	No sites	160, 266, 296, 329	N/A	160, 266, 296, 329
*T. subterraneus*	43, 303	No sites	No sites	N/A	43, 303

Analyses were performed simultaneously on all species and separate clades shown on Fig. 3. *N/A* not analyzed, *no sites* analysis did not show sites with a significant difference, *Manual* manual searching of sites with higher substitution density, *AA patterns* amino acid substitution patterns at intraspecific level

Despite programs giving different results in many cases, there are several sites consistent in several analyses: site 241 was indicated both with FEL and manual search on full-species dataset and on separate clades with *Ellobius sp.* (FEL) and *L. mandarinus* (PAML). Position 329 was detected by FEL in *L. mandarinus* and *P. schaposchnikowi* clades. Site 238 indicated by PAML and MEME analysis in *L. mandarinus* clade. All these sites (238, 241, and 329) show a significant difference in amino acid patterns.

**Fig. 4:**
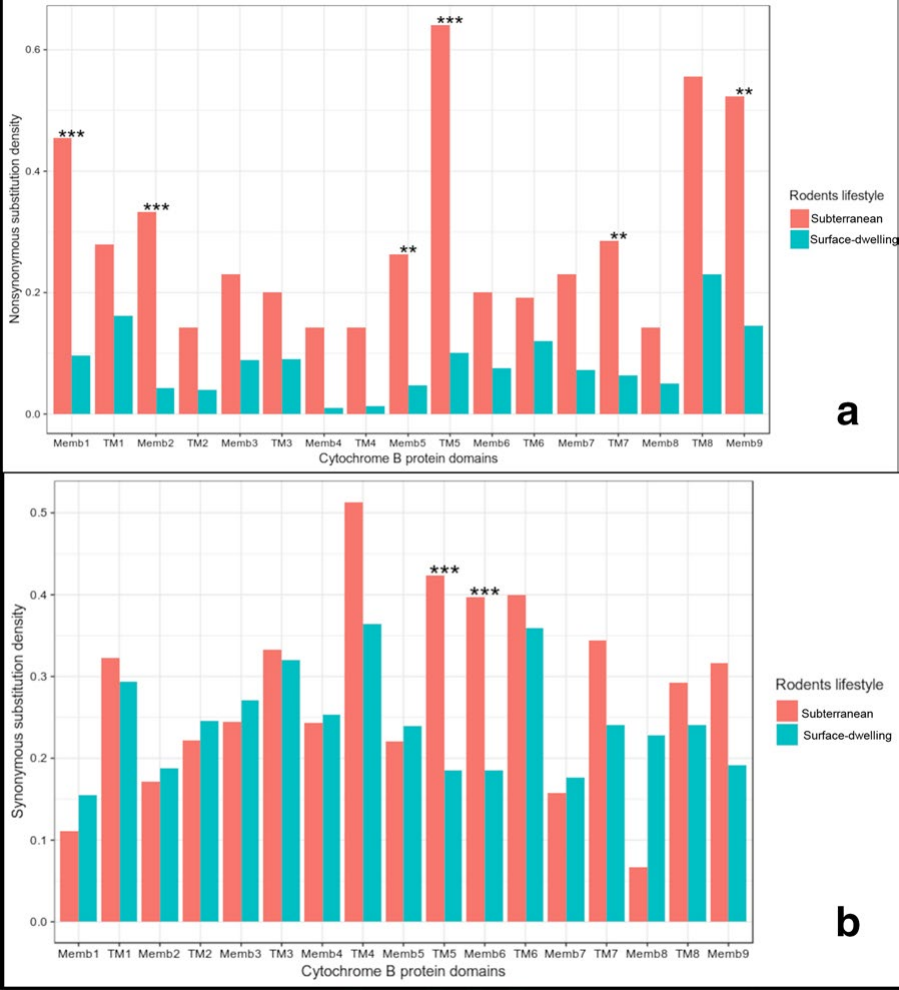
Domains in cytB gene with substitution frequency in subterranean and surface-dwelling species. *Memb* membrane domain, *TM* transmembrane domain. Stars indicate levels of significance. **p < 0.1, ***p < 0.001 **a** Nonsynonymous substitutions distribution across domains. **b** Synonymous substitutions

Comparison of substitutions in whole domains (Table 3, Fig. 4) uncovered significant differences in membrane domains 1, 2, 5, and 9 and transmembrane domains 5 and 7 for nonsynonymous substitutions (Fig. 4a); and membrane domain 6 and transmembrane domain 5 for synonymous substitutions (Fig. 4b). Its visualization (Additional file 2a) shows that the location does not correlate with special positions relative to other domains or complex components.

**Table 3:** Domains in *cytB* gene with a significant difference in substitution frequency between subterranean and surface-dwelling species

Domains	Subterranean	Surface-dwelling	Substitutions	Fs	Holm correction
Memb1	0.45	0.097	NS	0.00000033	0.000011
Memb2	0.33	0.043	NS	0.00001176	0.000365
Memb5	0.263	0.047	NS	0.00033176	0.008957
Memb9	0.52	0.15	NS	0.00005434	0.001522
TM5	0.64	0.10	NS	0.00000001	0.000000
TM7	0.29	0.06	NS	0.00004166	0.001208
Memb6	0.40	0.19	S	0.00000012	0.000004
TM5	0.42	0.19	S	0.00002733	0.000820

*NS* nonsynonymous substitutions, *S* synonymous substitutions, *Fs* Fisher exact test, *Holm correction* p-values after Holm-Bonferroni correction, *Memb* membrane domain, *TM* transmembrane domain

## Discussion

Purifying selection is a predominant force in the evolution of mtDNA. But it is possible that weak and/or episodic positive selection occurs simultaneously during the shift to a lifestyle with greater energy demands or reduced availability of oxygen [23, 24]. We examined the possibility of it in subterranean lineages bearing in mind that colonization of the subterranean niche results in exposure to environmental changes that may affect the function of mitochondrial genes. Among Arvicolinae, several independent, relatively recent, and non-simultaneous colonization of the subterranean niche can be examined for features consistent with convergent evolution under similar directional selection, using their non-subterranean relatives for comparison.

By implementing the standard tests we show that (1) several phylogenetically distant subterranean species show similar amino acid substitutions in cytochrome *b*, and these substitutions are plausibly important for the protein complex structure, (2) *cytB* showed an increased ratio between non-synonymous and synonymous substitutions (ω) in subterranean lineages compared to non-subterranean with evidence that selection strength has been relaxed, and (3) eight protein domains possess increased substitution ratio in subterranean species as well several nucleotide positions. Taken together, these results are consistent with the hypothesis that colonization of the subterranean niche promotes a new selective regime of positive, directional selection in protein-coding mtDNA genes. Below we discuss these findings to compare our results with previous findings on subterranean and surface-dwelling species from other taxa.

### Amino acid substitutions in *cytB* and their impact on the protein structure

Subterranean and surface-dwelling species of Arvicolinae were examined for the presence of amino acid substitutions in the mitochondrial *cytB* that could separate both. When only one sequence per species was used in TreeSAAP analysis, three sites with similar amino acid substitutions in distantly related subterranean species compared to all of the analyzed non-subterranean species were found, particularly Ser57Pro, Asp214Asn, and Ile338Val (Fig. 1). Using the results reported in [5], we compared substitution patterns in subterranean species from different families. A substitution at site 57 was also detected in African mole rats (family Bathyergidae), and in 214 in African mole rats and tuco-tucos (genus *Ctenomys*). An analogous substitution at site 214 was found to be under positive selection in high-altitude subterranean zokors *Eospalax fontanierii* Milne-Edwards, 1867 [32]. It is remarkable that among subterranean voles, substitution Asp214Asn was found in *Prometheomys schaposchnikowi, Ellobius fuscocapillus* and *Ellobius lutescens* and the same amino acid substitution was found in most of the specialized subterranean rodent families (Fig. 1). Among the Arvicolinae, the three mentioned species referred to the lineages with relatively earlier shifts and longer evolution periods compared to other subterranean forms in the subfamily. *Prometheomys schaposchnikowi,* the oldest lineage within the subfamily, represents the first wave of species radiation within Arvicolinae and is sister to all other recent lineages. The molecular time estimate for these split dates to approximate 7 Ma [33]. The putative origin of Ellobiusini is estimated as ca. 4 Ma, and other subterranean arvicolids, *T. subterraneus, M. pinetorum* and *L. mandarinus* belonging to the crown lineages within the Arvicolini tribe could not be earlier than 2 Ma. Thus, the occurrence of the same substitution in highly specialized species that originated at 2–5 Ma intervals, may indirectly indicate the importance of this substitution for adaptation to subterranean life. Also, the substitution at site 57 from serine to proline removes the opportunity for phosphorylation. No substitution was detected at site 338 in Spalacidae or Bathyergidae families but could be associated with the cancer process according to the ClinVar database. The latter may confirm the importance of substitution, but its impact and adaptive significance remain unclear.

The wide distribution of *cytB* as a phylogenetic marker allowed us to test changes in amino acid usage across the full-length protein. We detected more than 80 positions with significant changes including part of TreeSAAP detected positions. Amino acids Pro and Val are not major in positions 57 and 338, respectively, but used more often. Our models of the structure of *cytB* suggest that the substitution Ser57Pro can alter the dimerization of the complex since it is located on a loop segment that resides on the interface of the two *bc*_*1*_ monomers (Fig. 2). The functional role of the substitutions Asp214Asn and Ile338Val is less clear. The loop spanning residues 54–60 is rich in residues that potentially can form hydrogen bonds: Ser57, Asp58, Thr59, Thr60, Thr61 in *Lemmus sibiricus*. The Ser57Pro reduces the number of available hydrogen bonds, potentially weakening the interaction between loops. Additionally, this substitution eliminates a putative phosphorylation site, which can modulate the inter-loop interaction.

Both residues 214 and 338 of cytochrome *b* are in the vicinity of UQCRQ. In our model of *Lemmus sibiricus* Kerr, 1792, Asp214 forms an ion pair with an Arg2 of UQCRQ. This UQCRQ residue is conserved across multiple rodents (NCBI ID: RLQ55034.1, AAH28519.1, NP_777230.1, NP_001020305.1), and experimentally obtained structures from different mammals also display this interaction [32, 34]. Curiously, the Asp214Asn substitution in *E. lutescens* no longer interacts with Arg2 of UQCRQ; however, a co-occurring substitution—Asn212Asp—forms the ion pair instead of Asn214. The Ile338Val substitution may alter the binding of UQCRQ indirectly, through Ile350. Greater insight into the function of these substitutions may come with the gene sequences of UQCRQ for surface-dwelling and subterranean rodents. Taken together, structural modeling suggests the altered dimerization of cytochrome *bc*_*1*_ and UQCRQ binding.

### Signs of positive selection in the *cytB* gene

As OXPHOS is a conservative mechanism that is essential for energy metabolism, purifying selection dominates the evolution of mtDNA [24]. MtDNA-encoded *COX* subunits and *cytB* are the most conserved genes in mitochondrial genomes [29]. Due to the functional importance of mitochondrial genes, purifying selection is the dominant force in their evolution; however, weak and/or episodic positive selection may occur in the background of strong purifying selection if selective pressures shift, as might happen when oxygen availability decreases. Given the drastic change in energy requirements [2] and the transition from oxygen-rich to a hypoxic subterranean habitat [3–5], it is likely that genes involved in respiration experienced positive directional selection. Under this hypothesis, accelerated rates of functional (nonsynonymous) substitutions, relative to silent substitutions in these genes, are expected in subterranean organisms [24].

Signatures of positive selection were identified in a series of branch analyses implemented for a subset consisting of a subterranean species and a group of sisters surface-dwelling taxa and on the complete dataset. Both in comparing four *Ellobius* species against *Arvicola, Eothenomys* and *Chionomys* (Fig. 3) and *L. mandarinus* with other *Lasiopodomys* species and *Neodon* the ω-ratio was found to be significantly different between the foreground branch of subterranean species against the group of non-subterranean taxa. A significant difference can be observed by analyzing *T. subterraneus* against other *Terricola* and *Microtus* species, but the dN/dS ratio is conversely less for subterranean *T. subterraneus* compared with surface-dwelling. Surprisingly, comparison of *P. schaposchnikowi* against species of first Arvicolinae radiation (*Ondatra, Dicrostonyx, Myopus,* and *Phenacomys*) and *M. pinetorum* with other *Microtus* did not yield a significant difference between ω-values. When ω-values are smaller than one, the study of a single gene does not allow us to reject alternative, non-selection explanations for the rate variation, such as a relaxation of purifying selection, variations in metabolic rate, body mass, population size, and generation time between lineages [35–37]. However, Da Silva et al. [5] found a significantly higher ω in the *cytB* among phylogenetically distant subterranean family-level taxa of rodents (tuco-tucos, coruros, pocket gophers, and mole rats) compared to their above-ground relatives, suggesting a link between positive directional selection in this gene in subterranean lineages. Both the data of RELAX analysis consistent with codeml branch model results and show many sites under positive selection favor the latter suggestion.

Interestingly, the same adaptations in *COX* and *cytB* genes were linked to increased evolutionary rates in simian primates [38, 39] and in mammalian species adapted to unusual oxygen requirements [21, 23, 29]. This confirms the adaptive property of the *cytB* gene. The fact that we could not observe this difference for all species with subterranean lifestyle and less level of ω for *T. subterraneus* than for phylogenetically close non- subterranean species sites may be related to less pronounced fossorial in the latter species than in highly specialized subterranean forms such as *Ellobius* and *L. mandarinus.*

### Substitution density across *cytB*

We examined the substitution frequencies by domains in subterranean and surface-dwelling rodents. Significant differences in frequencies were observed more often with nonsynonymous substitutions than with synonymous ones. This result is consistent with our data on dN/dS estimations (Fig. 4).

According to several studies [40–42], the three main structural domains of the *cytB* protein are characterized by pronounced differences in the levels of amino acid variation. Gering et al. [42] consider these to be an adaptation to high-altitude in *Peromyscus maniculatus,* as the matrix domain was the most variable and the intermembrane domain was the least variable. In the taxa that were studied and described here, we came across the opposite situation: from six domains with significant differences in nonsynonymous substitutions, four belong to the membrane (intermembrane) domains and only two to the transmembrane domains. The influence of nonsynonymous substitutions on the protein structure is unclear due to a substitution-compensating mechanism. However, the great difference in substitution ratio (especially nonsynonymous) provides evidence in favor of the relaxation of purifying selection revealed with ω estimations.

## Conclusions

Our results indicate the signatures of positive selection in the evolution of mitochondrial DNA in Arvicolinae during colonization of subterranean environments. We observe relaxation of selection in *cytB* sequence using dN/dS calculation with branch, branch-site, and site models. Also, we detect significant differences in substitution distribution by domain structure and changes in amino acid usage for underground rodents. On top of this, we found similar amino acid substitution among phylogenetically distant subterranean lineages that could affect protein structure. Our data corroborate the recent findings that suggest that the evolution of mitochondrial protein genes, in particular *cytB,* could be associated with metabolic adaptations to environments with low oxygen availability.

## Methods

### Samples

In our analysis, we used 62 *cytB* sequences from Arvicolinae species that represent all major genera and tribes. Among these, several phylogenetically independent lineages including those adapted to existence in the subterranean environment. Here, we consider all species of the genus *Ellobius*, monotypic *Prometheomys schaposchnikowi*, and the species *Lasiopodomys mandarinus, Terricola subterraneus* and *Microtus pinetorum* as subterranean. We compared these taxa with non-subterranean representatives of 22 genera: *Alexandromys, Alticola, Arvicola*, *Blanfordimys*, *Chionomys*, *Clethrionomys, Craseomys, Dicrostonyx, Eolagurus, Eothenomys, Lagurus, Lasiopodomys, Lemmus, Microtus*, Myopus, *Neodon*, *Neofiber*, *Ondatra*, *Phenacomys*, *Synaptomys*, *Terricola, Volemys* and used *Cricetulus, Mesocricetus, Peromyscus* and *Phodopus* as an outgroup. The full list of species and GenBank accession numbers are given in Additional file 3.

### DNA extraction, amplification and sequencing

Muscle and skin tissue samples were collected between years of expeditions and stored in 96% ethanol at − 20 degrees Celcius in a tissue and DNA collection of the Group of molecular systematics of mammals (Zoological Institute RAS). Genomic DNA was isolated from ethanol-preserved muscle tissues using a standard salt extraction protocol [43]. For the better resolution of the Arvicolinae tree, alongside mitochondrial *cytB*, we used and seven nuclear genes: breast cancer 1 gene (*BRCA1*), exon 11; growth hormone receptor gene (*GHR*), exon 10; a fragment of the lecithin cholesterol acyltransferase gene (*LCAT*), exons 2–5 and introns 2–4; tumor suppressor protein gene (*TP53*), exons 5–7 and introns 5–6; interphotoreceptor retinoid-binding protein gene (*IRBP*); von Willebrand factor gene (*vWF*), exon 28; and acid phosphatase type V gene (*Acp5*), exons 2 and 3 and partial coding sequence, were amplified. All the primers used and references where the PCR conditions are given are listed in Table 4. All sequences obtained in this work are marked with bold in Additional file 3.

**Table 4:** Primers used in the study

Genes	Primer names	Primer sequence (5′–3′)	Reference
*cytB*	*L14729*	*GACATGAAAAATCATCGTTGTTATT*	[44]
	*H15985*	*TAGAATGTCAGCTTTGGGTGCT*	[45]
*BRCA1*	*F180_arv*	*CGGAACAGATGGGCTGAAAGTAAAG*	[46]
	*R1240_arv*	*GGCATCTGCTGCAGGTTCTGTGT*	
*GHR*	*arv_F*	*GGCGTTCATGACAACTACAAACCTGA*	[9]
	*arvic_R*	*ATAGCCACACGAGGAGAGGAACT*	
*LCAT*	*LCAT F*	*CACCATCTTCCTGGATCTCAA*	[9]
	*LCAT R*	*AAGAAATACAGCACATGTAGGCA*	
*TP53*	*p53 2F*	*TYCCCTCAATAAGCTRTTCTGCCA*	[47]
	*p53 3R*	*GTTTATGCCCCCCATGCAGA*	
*IRBP*	*A3*	*CTGATGGGAATGCAAGCAGC*	[47]
	IPL*	*GACATCGCCTACATCCTCAAGCA*	
	IPR*	*CTCAGCTTCTGSAGGTCYAGG*	
	*B2a*	*ATGAGGTGYTCYGTGTCCTG*	
*vWF*	*V1'*	*TGTSAACCTYACSTGTGAAGCCTG*	[48]
	VIF*	*CTACCTCTGTGACCTTGCCCCTGA*	
	VIR*	*TCAGGGGCAAGGTCACAGAGGTAG*	
	*W1*	*TGCAGGACCAGGTCAGGAGCCTCTC*	
*Acp5*	*AP5-120fwd*	*AATGCCCCATTCCACACAGC*	[10]
	*AP5-564rev*	*CCCGGGAAATGGCCAATG*	

Internal primers for sequencing are marked with an asterisk (*)

PCR cleanup was performed using the Omnix kit (Omnix, Russia). PCR products were sequenced in both directions using ABI BigDye version 3.1. Sequences were edited and aligned with Geneious R11 (https://www.geneious.com), we also checked that sequences obtained were coded correctly. Final alignments had the following lengths: *cytB* 1143 bp, *BRCA1* 1022 bp, *GHR* 869 bp, *LCAT* 607 bp, *TP53* 949 bp, *IRBP* 1267 bp, *vWF* 1251 bp, and *Acp5* 454 bp. The sequences obtained in the current study were deposited in GenBank (Additional file 3).

### Phylogenetic reconstruction

The full list of genes and GenBank accession numbers used are provided in Additional file 3. The best-fit of several substitution models for each gene (TVM:G:5 for Acp5, GTR:G:5 for BRCA1, HKY:G:5 for GHR, TN:G:5 for TP53, J3:G:5 for LCAT, J2:G:5 for IRBP and vWF) was assessed using Treefinder [49] under the corrected Akaike information criterion (AICc). Bayesian analysis based on the concatenated alignment of seven genes (partitioned by gene) was performed in MrBayes 3.2.6 [50]. Each analysis started with random trees and two independent runs with 4 Markov chains Monte Carlo (MCMC) were performed for 5 million generations, with sampling every 1000th generation; the standard deviations of split frequencies were below 0.01, potential scale reduction factors were equal to 1.0. Stationarity and convergence of separate runs was examined in Tracer v1.6 [51]. A consensus tree was constructed based on the trees sampled after the 25% burn-in.

### Amino acid substitutions detection

Significant physicochemical amino acid changes between residues in *cytB* were identified using the modified MM01 model implemented in TreeSAAP v3.2 [30]. Eight categories (1–8) were used to represent the magnitude of radical substitutions, of which categories 6–8 indicate the most radical substitutions, by setting a sliding window of 20 codons and analyzing the properties of 31 amino acids [52]. Significant positive z-scores (categories 6–8, P < 0.001) were accepted as a sign of significant change in function.

The distribution of synonymous and nonsynonymous substitutions was calculated between subterranean and surface-dwelling species in each site separately and combined by domain coordinates. Domain coordinates used accordingly UniProt information of Mus musculus cytochrome b domains: https://www.uniprot.org/uniprot/P00158.

Amino acid patterns for each position were determined using all sequences for selected species to consider intraspecific variation. Altogether more than 6 200 sequences were analyzed: 131 for subterranean species and 6 059 for surface-dwelling. This dataset includes all available in Genbank *cytB* sequences in August 2020. Amino acid patterns were calculated using the script on Python 3. Statistical test was selected considering unequal sample sizes for all analyses.

The significance of the substitution frequency and amino acids patterns were estimated using Fisher’s exact test and Holm multiple adjustment. All computations were performed in R software v.3.4.4 [53].

### Synonymous and nonsynonymous substitution estimations

Variation in the estimates of dN, dS, and ω (dN/dS ratio) was explored using the codeml approach, as implemented in ete-toolkit [54] For each subterranean species (or group of species for *Ellobius*) branch analysis was implemented with free-branch model (b_free, were ωfrg and ωbkg are free), neutral-branch model (b_neut, were ωfrg is fixed to one) and M0 model, where all branches evolve at the same rate. Subdivision into analyzed groups was carried out according to the principle of selection of phylogenetic nearest surface-dwelling taxa for ω comparison and more distant as outgroup. Were calculated likelihood-ratio tests to compare different models. The comparison between free-branch and M0 showed if foreground branches have an ω significantly different from the rest of the tree. And the comparison between free-branch and neutral-branch models detect if the value of ωfrg is significantly higher than 1.

Several programs from DataMonkey web-server (datamonkey.org) was used for search selection signatures: aBSREL (An adaptive branch-site REL test for episodic diversification [55]), FEL (Fixed Effects Likelihood [56]) and MEME (Mixed Effects Model of Evolution [57]). Also, we performed PAML [58] site analysis with M2 model for looking sites under positive selection [59].

### Modelling

We modeled homology-based structures of cytochrome *b* as part of the *bc*_*1*_ complex from *L. sibiricus* and *E. lutescens*. The overall architecture of the *bc*_*1*_ complex varies little in crystal structures from different organisms ranging from yeast to several mammalian species [60, 61], justifying homology modeling. We based the model on the crystal structure and protein sequence of cytochrome *bc*_*1*_ complex from *Bos taurus* with a resolution of 2.4 Å (Protein Data Bank ID: 1NTM [32]). First, the homodimeric structure was recovered by exploiting the symmetry of the crystallographic group. Next, we used modeller (release 9.22) [62] to create structures of the *bc*_*1*_ homodimer by using the sequence of the cytochrome *b* from the corresponding organism but keeping the other parts of the complex from *Bos taurus*. We adopted the auto model protocol with default settings. The models were superimposed to 1NMT and substitutions were analyzed visually in PyMOL v.2.0 (Schrödinger, LLC), which was also used to produce figures. Transmembrane regions of the complex were estimated using the OPM web server [63].

### Phosphorylation prediction

To predict phosphorylation sites, we used two different tools—NetPhos 3.1 Server (http://www.cbs.dtu.dk/services/NetPhos/) [64] and GPS 5.0 (http://gps.biocuckoo.cn/online.php) [65].

## Supplementary Information

**Additional file 1.** Amino acid sites with significant difference between subterranean and surface-dwelling rodents.

**Additional file 2.** Analyzed position visualization. A. Domains with a significantly increased frequency of nonsynonymous substitutions. B Sites with significant changes in amino acid usage.

**Additional file 3.** Species and genes used in the study. Sequences received in this work marked bold.

## Data Availability

All genes, used for alignment, are available in GenBank, IDs are provided in Additional file 3. All primers sequences used for gene amplification located in Table 4.

## Abbreviations

*cytB*: Cytochrome *b* gene
Ma: Million years ago
MRCA: Most recent common ancestor
dN: Amount of nonsynonymous substitutions
dS: Amount of synonymous substitutions
ω: The ratio of nonsynonymous to synonymous substitutions
CDC2: Cell division control 2 kinase
AGC: Aggrecan kinase
PKN: Protein kinase N
PKN1: Protein kinase N1
AICc: Corrected Akaike information criterion
MCMC: Markov chains Monte Carlo
*BRCA1*: Breast cancer 1 gene
*GHR*: Growth hormone receptor gene
*LCAT*: Lecithin cholesterol acyl transferase gene
*TP53*: Tumor suppressor protein gene
*IRBP*: Interphotoreceptor retinoid-binding protein gene
*vWF*: Von Willebrand factor gene
*Acp5*: Acid phosphatase type V gene
mtDNA: Mitochondrial DNA
LRT: Likelihood-ratio test
UQCRQ: Ubiquinol-cytochrome c reductase complex III subunit VII
