# Supplementary figures and images for: Searching for signatures of positive selection in cytochrome b gene associated with subterranean lifestyle in fast-evolving arvicolines (Arvicolinae, Cricetidae, Rodentia)
Source: BMC Ecol Evol. 2021 May 20;21:92. doi: 10.1186/s12862-021-01819-4 (PMC8136191; doi:10.1186/s12862-021-01819-4)

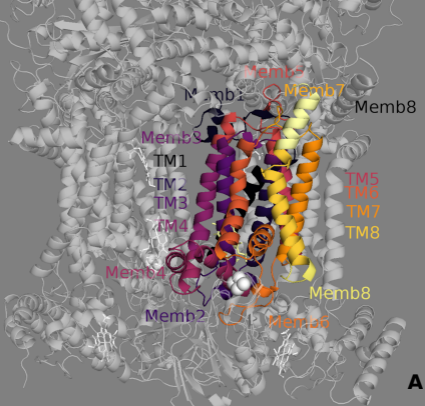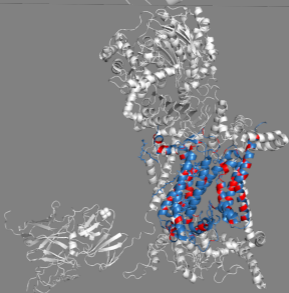

Supplement: Supplementary file 2 — Additional file 2. Analyzed position visualization. A. Domains with a significantly increased frequency of nonsynonymous substitutions. B Sites with significant changes in amino acid usage. [file 12862_2021_1819_MOESM2_ESM.pdf]
